# Dynamic properties of successful smiles

**DOI:** 10.1371/journal.pone.0179708

**Published:** 2017-06-28

**Authors:** Nathaniel E. Helwig, Nick E. Sohre, Mark R. Ruprecht, Stephen J. Guy, Sofía Lyford-Pike

**Affiliations:** 1 Department of Psychology, University of Minnesota, Minneapolis, MN, United States of America; 2 School of Statistics, University of Minnesota, Minneapolis, MN, United States of America; 3 Department of Computer Science & Engineering, University of Minnesota, Minneapolis, MN, United States of America; 4 Department of Otolaryngology-Head and Neck Surgery, University of Minnesota, Minneapolis, MN, United States of America; University of Portsmouth, UNITED KINGDOM

## Abstract

Facial expression of emotion is a foundational aspect of social interaction and nonverbal communication. In this study, we use a computer-animated 3D facial tool to investigate how dynamic properties of a smile are perceived. We created smile animations where we systematically manipulated the smile’s angle, extent, dental show, and dynamic symmetry. Then we asked a diverse sample of 802 participants to rate the smiles in terms of their effectiveness, genuineness, pleasantness, and perceived emotional intent. We define a “successful smile” as one that is rated effective, genuine, and pleasant in the colloquial sense of these words. We found that a successful smile can be expressed via a variety of different spatiotemporal trajectories, involving an intricate balance of mouth angle, smile extent, and dental show combined with dynamic symmetry. These findings have broad applications in a variety of areas, such as facial reanimation surgery, rehabilitation, computer graphics, and psychology.

## Introduction

The ability to express emotional intent via facial expression is a foundational aspect of social interaction and nonverbal communication. Since Paul Ekman’s pioneering work [1–3], much effort has been devoted to the study of the emotional processing of facial expressions. Research has revealed that a variety of different emotional cues can be perceived within 100 to 200 ms after encountering a face [4, 5]. Furthermore, studies have shown that evaluations of facial emotions can have immensely important societal outcomes, e.g., recognizing an angry face to avoid a threat or recognizing a trustworthy face to determine a good leader [6]. The smile is the most well-studied facial expression, given that smiles are used frequently during interpersonal interactions [7]. Previous research suggests that an inability to effectively smile increases one’s risk for depression [8], which highlights the smile’s important role in mental health.

Unfortunately, tens of thousands of individuals each year suffer from trauma, cerebrovascular accidents (strokes), neurologic conditions, cancers, and infections that rob them of the ability to express emotions through facial movement [9]. The psychological and social consequences are significant. Individuals with partial facial paralysis are often misinterpreted, have trouble communicating, become isolated, and report anxiety, depression, and decreased self-esteem [8, 10, 11]. One option for such individuals is known as facial reanimation, which consists of surgery and rehabilitation aimed at restoring facial movement and expression. Despite the prevalence of facial reanimation procedures, clinicians lack rigorous quantitative definitions of what constitutes a socio-emotionally effective facial expression [12–14]. The key question is this: what spatial and temporal characteristics are most pertinent for displaying emotions in a real-world (dynamic) setting?

While much has been discovered about the psychology of facial expression and the perception of emotions, less is known about the impact of dynamic elements, e.g., the rate of mouth movement, left-right asymmetries, etc. This is largely because the vast majority of facial expression studies have been conducted on static images of actors expressing different facial emotions, thereby ignoring the temporal component associated with these expressions. In real-world applications, the dynamics of a facial expression can drastically influence facial perception [5, 15, 16]. Successfully treating patients who have facial movement disorders fundamentally requires an adequate understanding of both the spatial and temporal properties of effective emotional expression. However, the temporal components of facial emotional perception are less frequently studied, because systematically manipulating the timing of emotional expressions is a very difficult task—even for the best-trained actor.

Synthetic models of human facial expressions [16–18] offer exciting possibilities for the study of spatial and temporal aspects of dynamic expressions of emotion. Past research has revealed that spatial and temporal characteristics of dynamic facial expressions can be useful for distinguishing between different types of smiles [19, 20]. Furthermore, some studies have shown that the dynamics of facial expressions can have important, real-world economic and social outcomes [21, 22], and other studies have examined the role of symmetry (or asymmetry) in dynamic facial expressions [23, 24]. The general consensus is that dynamic aspects of facial expressions can have noteworthy affects on the perception of the expression, and more work is needed to understand how subtle spatiotemporal changes of facial expressions alter their intended meaning.

In this work, we leverage recent advances in computer animation and statistical learning to explore the spatiotemporal properties associated with a successful smile. Specifically, we develop a 3D facial tool capable of creating dynamic facial expressions, which allows us to isolate and manipulate individual features of lip motion during a smile. The resulting tool is able to control the timing of a smile to a greater degree than is possible with trained actors, and allows us to manipulate clinically relevant features of smiles. Using this tool, we investigate which combinations of spatial (i.e., mouth angle, smile extent, and dental show) and temporal (i.e., delay asymmetry) parameters produce smiles judged to be “successful” (i.e., effective, genuine, and pleasant) by a large sample of fairgoers (802 participants).

For this study, we focused on analyzing only the effect of mouth motion, given that (i) smiling impairment due to restricted mouth motion has been specifically shown to increase depressive symptoms in patients with facial neuromuscular disorders [8], and (ii) existing surgical interventions have shown particular success in rehabilitating corresponding muscles after trauma [9]. Although orbicularis oculi contraction is important in Duchenne smiles [7], to date, techniques are limited in restoring periocular movement. Multiple approaches focus on restoring mouth movement, so we seek to understand the effects of targeted manipulation of the mouth on the perception of smiling expressions. Past studies have found that the lower-half of the face (particularly the mouth shape) is the most salient factor for determining the intended meaning of a smile [25]. Thus, with this model, we expect the study participants to perceive differences in the emotions of the expressions and, as a result, provide meaningful information for clinical translation.

## Materials and methods

### Computer-animated facial tool

Using an interpolative blend shape approach [26], we developed a computer-animated, realistic 3D facial tool capable of expressing a variety of emotions. Similar to other recent anatomically motivated face simulation systems such as FACSgen [17] and FACSgen 2.0 [18], our model follows linear motion interpolation between anatomically valid static face poses. The modeling process was closely monitored and rechecked by a board-certified facial reconstructive surgeon (coauthor Lyford-Pike) in order to ensure a high degree of anatomical accuracy of the resulting mouth animation. Importantly, our model allows us to manipulate the character’s mouth independently of other muscle groups. This allows us to focus our study directly on mouth motion, which is both one of the most important aspects for visually identifying emotion [25] and the element of face movement most easily manipulatable through surgical intervention. The resulting face generation model supports variations in the extent, dental show, position, angle, timing, and asymmetry of mouth motion.

**Fig 1:**
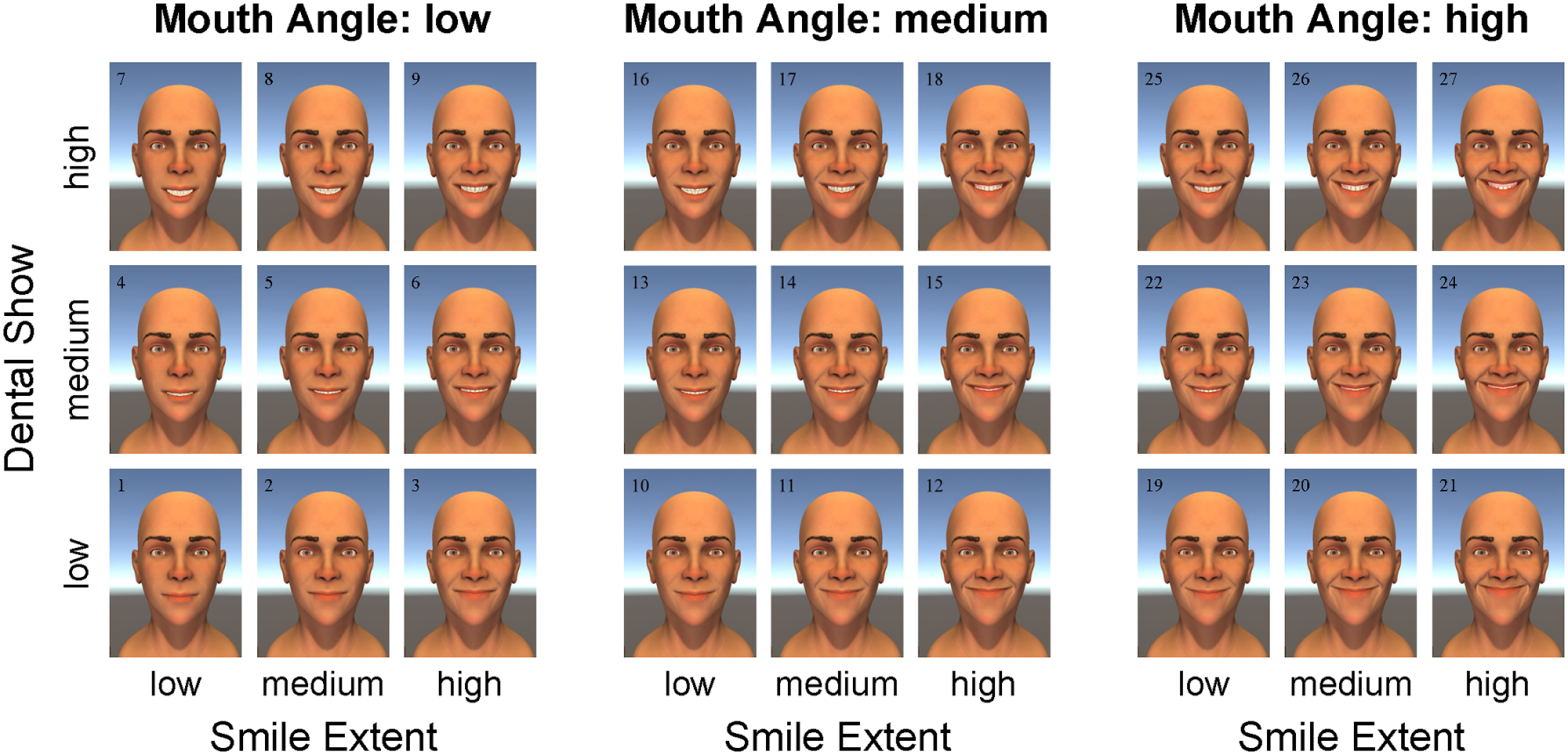
The 27 different smiling faces. The 27 smiles represent all possible combinations of the three spatial factors (mouth angle, smile extent, dental show) at three different levels (low, medium, high). The numbers 1–27 have been included post hoc for labelling purposes and were not present in the animations.

**Fig 2:**
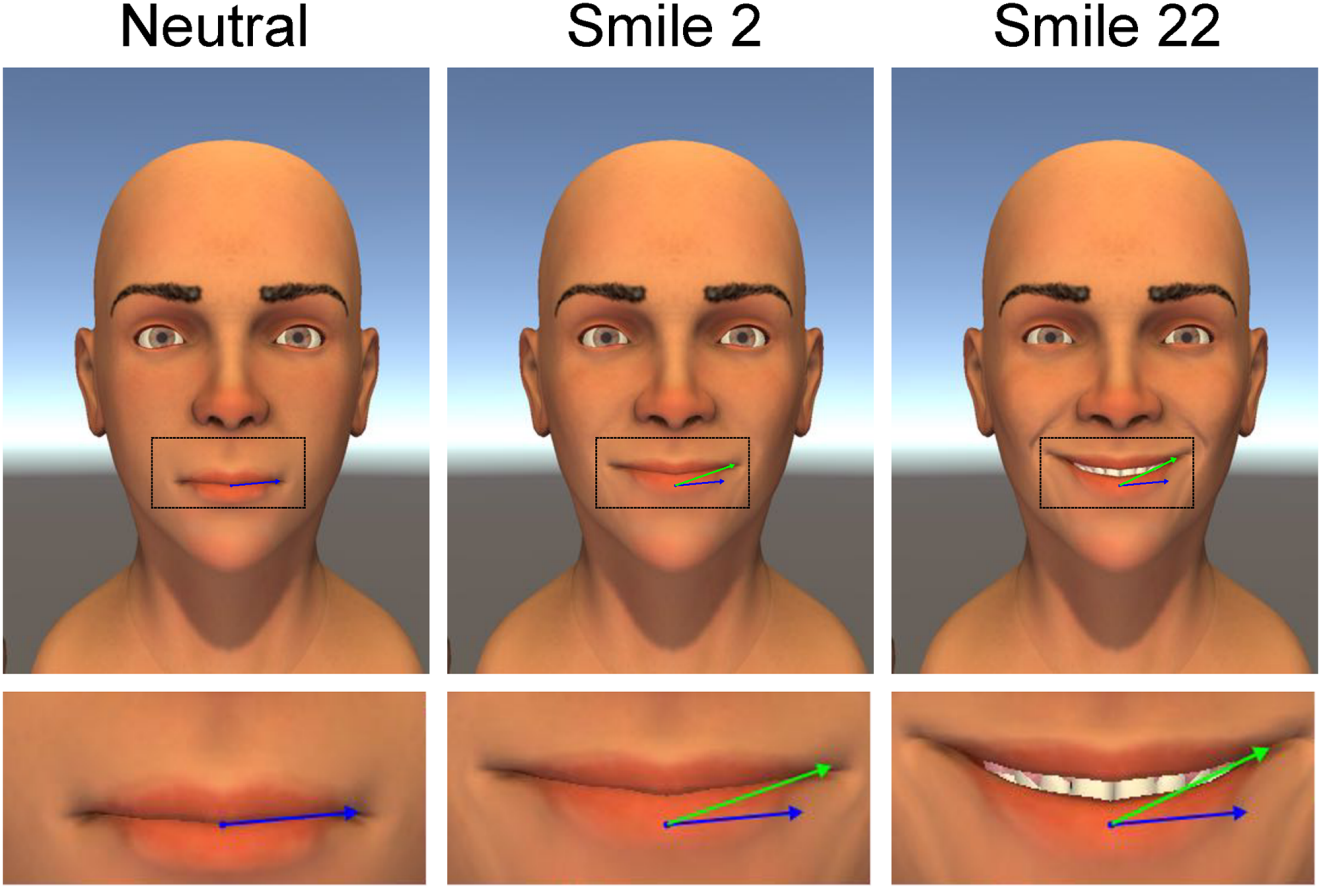
Definitions of the spatial parameters used in the study. Mouth angle is the angle between the green and blue lines. Smile extent is the length of the green line. Dental show is the distance between the lower and upper lips.

**Fig 3:**
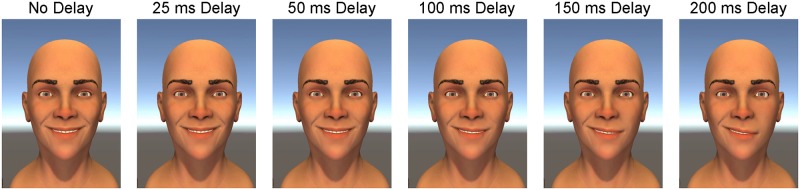
Smile 22 with various amounts of timing (delay) asymmetry. All animations started with the same (symmetric) neutral expression and ended with the same (symmetric) smiling expression, so the asymmetries were only visible for a few frames of the 250 ms animation.

With this tool, we created 250 ms animations of smile-like expressions, systematically manipulating spatial and temporal properties. We focus on 27 stimuli (see Fig 1) that were created by taking a systematic sweep of three blend shapes. The three blend shapes were designed to manipulate three parameters: (i) the mouth angle, (ii) the smile extent, and (iii) the amount of dental show (see Fig 2). Before collecting data, we designated smile 22 (high mouth angle, low smile extent, and medium dental show) as a prototypical smile for the investigation of timing asymmetries. To create spatiotemporal asymmetries in the smiling expressions, we manipulated the timing delay of the left side of the facial expression for smile 22. In addition to the symmetric versions of smile 22 previously described, we created five other versions of smile 22 with different delay asymmetries (see Fig 3). The delay asymmetries were created by delaying the start of the smile expression on the left side of the face.

### Study participants

**Fig 4:**
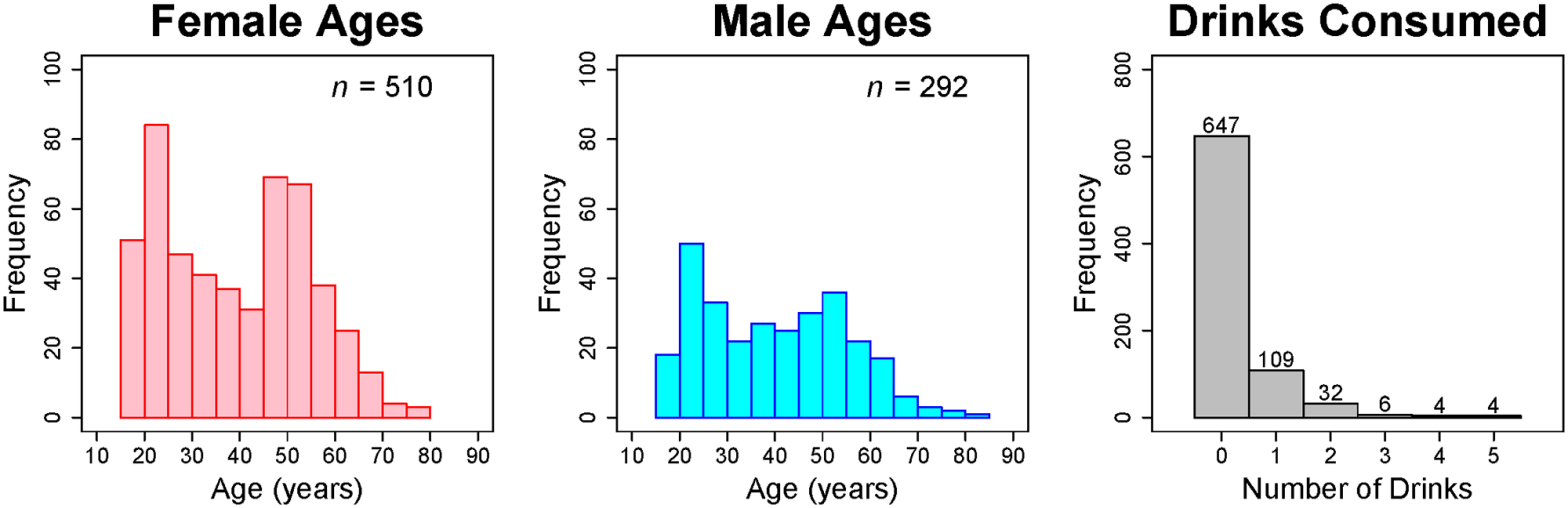
Histograms of demographic variables. Age distributions for female (left) and male (middle) participants, as well as the alcohol consumption numbers (right).

We use data collected from a diverse sample of study participants over the course of three days at the 2015 Minnesota State Fair in the University of Minnesota’s Driven to Discover building. Note that using fairgoers as the “general public” should provide a more representative sample compared to the WEIRD sample that is commonly used in behavioral research [27]. Participants ranged in age from 18 to 82, and there was a bimodal age distribution for both the female and male participants with peaks at about 20 and 50 years of age, see Fig 4. Participants were excluded from our analyses if they (i) had consumed six or more alcoholic drinks that day, and/or (ii) did not complete the entire survey. Our final sample included 802 participants (510 females and 292 males) who met the inclusion criteria for our study.

### Procedure

**Fig 5:**
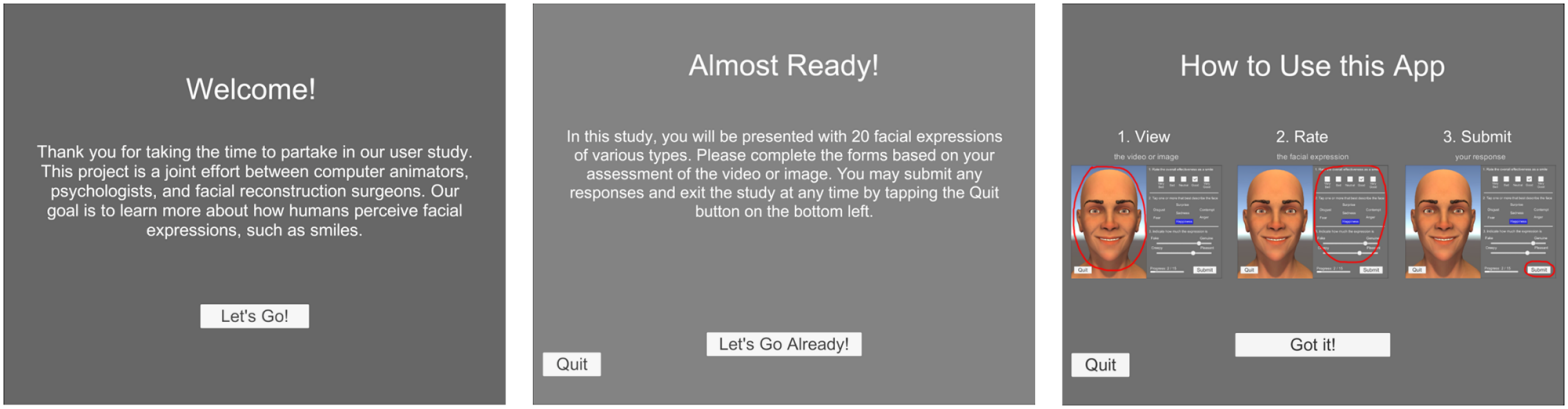
Screenshots from smile study iPad app. The welcome screen, consent screen, and instructions screen that were shown to participants at the onset of our study.

Our study protocol (including our informed consent procedure) was approved by the Institutional Review Board at the University of Minnesota. During the 2015 Minnesota State Fair, we asked laypersons who entered, and/or walked by, the Driven to Discover building to participate in our “Smile Study”. As compensation for participating in our study, participants were entered in a drawing to win an iPad. Upon verbally consenting to participate in our study, a volunteer explained to each individual that we were interested in how people perceived facial expressions of emotion. After the basic introduction, each participant was handed an iPad with a custom-built app, which contained a welcome screen, an information/consent screen, and an instructions screen (see Fig 5). After the instructions screen, participants provided some basic demographic information: age, gender, zip code, and number of alcoholic drinks they had consumed that day. Then each participant was shown 15 randomly sampled animations, followed by five still pictures of facial expressions of emotion. Note that the animations were randomly sampled from a larger population of facial expressions, but in this paper we only analyze the data corresponding to the 27 animations in Fig 1.

For each stimulus, participants were asked to (i) “Rate the overall effectiveness as a smile” using a 5-point Likert scale: Very Bad, Bad, Neutral, Good, and Very Good, (ii) “Tap one or more [words] that best describe the face” using a list of seven emotions: Anger, Contempt, Disgust, Fear, Happiness, Sadness, and Surprise, (iii) “Indicate how much the expression is” Fake (low end) versus Genuine (high end) using a continuous slider bar, and (iv) “Indicate how much the expression is” Creepy (low end) versus Pleasant (high end) using a continuous slider bar. Throughout the remainder of the paper, we refer to the ratings as (i) Effective ratings, (ii) Emotion ratings, (iii) Genuine ratings, and (iv) Pleasant ratings, respectively. Participants were instructed to interpret the words Effective, Fake, Genuine, Creepy, and Pleasant in a colloquial sense of these words. This was done to avoid biasing the participants’ opinions, so that the ratings could be interpreted in a colloquial—instead of a clinical—sense of these words. Participants were allowed to quit the study at any point.

### Data analysis

#### Overview

For the Genuine (Pleasant) ratings, the endpoints of the continuous slider bar were numerically coded as 0 = Fake (0 = Creepy) and 1 = Genuine (1 = Pleasant), so a score of 0.5 corresponds to a “neutral” rating—and scores above 0.5 correspond to above neutral ratings. Similarly, Effective ratings were numerically coded as 0 = Very Bad, 0.25 = Bad, 0.5 = Neutral, 0.75 = Good, and 1 = Very Good, so scores above 0.5 correspond to above neutral ratings. We define a “successful smile” as one that is rated above neutral in overall effectiveness, genuineness, and pleasantness. To determine the spatial and temporal properties related to a successful smile, we use a nonparametric mixed-effects regression approach [28–32]. More specifically, we used a mixed-effects extension of smoothing spline analysis of variance (SSANOVA) [33, 34]. The models are fit using the “bigsplines” package [35] in the R software environment [36]. Inferences are made using the Bayesian interpretation of a smoothing spline [37, 38].

#### Symmetric smiles

To determine how spatial (mouth angle, smile extent, and dental show) properties affect the perception of a smile, we use a nonparametric mixed-effects model of the form

$$\begin{array}{c} y_{ij}=\eta_{A}\left(a_{i}\right)+\eta_{G}\left(g_{i}\right)+\eta_{D}\left(d_{i}\right)+\eta_{S}\left(s_{ij}\right)+b_{i}+\epsilon_{ij} \end{array}$$ (1)

where *y*_*ij*_ is the rating that the *i*-th participant assigned to the *j*-th stimulus, *η*_*A*_(⋅) is the unknown main effect function for age with *a*_*i*_ ∈ {18, …, 82} denoting the age of the *i*-th participant, *η*_*G*_(⋅) is the unknown main effect function for gender with *g*_*i*_ ∈ {*F*, *M*} denoting the gender of the *i*-th participant, *η*_*D*_(⋅) is the unknown main effect function for drinking with *d*_*i*_ ∈ {0, 1, 2, 3, 4, 5} denoting the number of alcoholic drinks consumed by the *i*-th participant, and *η*_*S*_(⋅) is the unknown smile effect function with **s**_*ij*_ = (*s*_*ij*1_, *s*_*ij*2_, *s*_*ij*3_)′ denoting a 3 × 1 vector containing the known spatial properties of the *j*-th stimulus displayed to the *i*-th participant such that *s*_*ij*1_ ∈ {low, medium, high} denotes the mouth angle, *s*_*ij*2_ ∈ {low, medium, high} denotes the smile extent, and *s*_*ij*3_ ∈ {low, medium, high} denotes the amount of dental show. The unknown parameter *b*_*i*_ is a random baseline term for the *i*-th participant, which allows each participant to have a unique intercept term in the model. The *b*_*i*_ terms are assumed to be independent and identically distributed (iid) Gaussian variables with mean zero and unknown variance *θ*^2^. Finally, the *ϵ*_*ij*_ terms are unknown error terms, which are assumed to be (i) iid Gaussian variables with mean zero and unknown variance *σ*^2^, and (ii) independent from the *b*_*i*_ effects.

Using the SSANOVA model, the smile effect function *η*_*S*_ can be decomposed such as

$$\begin{array}{c} \begin{array}{cc} \eta_{S}\left(s\right) & =\eta_{0}+\eta_{1}\left(s_{1}\right)+\eta_{2}\left(s_{2}\right)+\eta_{3}\left(s_{3}\right)+\eta_{12}\left(s_{1},s_{2}\right)\\ & \;+\eta_{13}\left(s_{1},s_{3}\right)+\eta_{23}\left(s_{2},s_{3}\right)+\eta_{123}\left(s_{1},s_{2},s_{3}\right) \end{array} \end{array}$$ (2)

where *η*_0_ is an unknown constant, *η*_1_(⋅), *η*_2_(⋅), and *η*_3_(⋅) denote the main effect functions for the three spatial parameters (angle, extent, and dental show, respectively), *η*_12_(⋅) denotes the angle-extent interaction effect function, *η*_13_(⋅) denotes the angle-dental show interaction effect function, *η*_23_(⋅) denotes the extent-dental show interaction effect function, and *η*_123_(⋅) denotes the three-way interaction effect function. The model in Eq (2) includes all possible two-way and three-way interactions between the spatial parameters, but we could consider simpler models that remove some (or all) of the interaction effects. The simplest model has the form

$$\begin{array}{c} \eta_{S}\left(s\right)=\eta_{0}+\eta_{1}\left(s_{1}\right)+\eta_{2}\left(s_{2}\right)+\eta_{3}\left(s_{3}\right) \end{array}$$ (3)

which only contains the additive effects of the three spatial parameters. To determine which effects should be included in the model, we fit the nine possible models (see Table 1), and we used the AIC [39] and BIC [40] to choose the model that provides the best fit relative to the model complexity.

**Table 1:** Models for smile effect function *η*_*S*_.

#	*η*_*S*_
1.	*η*_0_ + *η*_1_ + *η*_2_ + *η*_3_ + *η*_12_ + *η*_13_ + *η*_23_ + *η*_123_
2.	*η*_0_ + *η*_1_ + *η*_2_ + *η*_3_ + *η*_12_ + *η*_13_ + *η*_23_
3.	*η*_0_ + *η*_1_ + *η*_2_ + *η*_3_ + *η*_12_ + *η*_13_
4.	*η*_0_ + *η*_1_ + *η*_2_ + *η*_3_ + *η*_12_ + *η*_23_
5.	*η*_0_ + *η*_1_ + *η*_2_ + *η*_3_ + *η*_13_ + *η*_23_
6.	*η*_0_ + *η*_1_ + *η*_2_ + *η*_3_ + *η*_12_
7.	*η*_0_ + *η*_1_ + *η*_2_ + *η*_3_ + *η*_13_
8.	*η*_0_ + *η*_1_ + *η*_2_ + *η*_3_ + *η*_23_
9.	*η*_0_ + *η*_1_ + *η*_2_ + *η*_3_

*Note*: 1 = angle, 2 = extent, 3 = dental show.

We fit the nine models in Table 1 using three different response variables: smile effectiveness, smile genuineness, and smile pleasantness. For each of the fit models, we used a cubic smoothing spline for the age and drinking marginal effects, a nominal smoothing spline (i.e., shrinkage estimator) for the gender effect, and an ordinal smoothing spline for the angle, extent, and dental show marginal effects [33]. The interaction effects are formed by taking a tensor product of the marginal smoothing spline reproducing kernels [34]. The models are fit using the two-step procedure described in [29], which uses a REML algorithm [41] to estimate the unknown variance component *θ*^2^ (step 1) followed by a generalized cross validation (GCV) routine [42] to estimate the unknown smoothing parameters (step 2).

#### Asymmetric smiles

To examine how timing asymmetry influences the interpretation of smile expressions, we analyzed six variations of smile 22 using a model of the form

$$\begin{array}{c} y_{ij}=\eta_{0}+\eta_{A}\left(a_{i}\right)+\eta_{G}\left(g_{i}\right)+\eta_{D}\left(d_{i}\right)+\eta_{T}\left(t_{ij}\right)+b_{i}+\epsilon_{ij} \end{array}$$ (4)

where *η*_*T*_(⋅) is the unknown main effect function for timing (delay) asymmetry with *t*_*ij*_ ∈ {0, 25, 50, 100, 150, 200} denoting the delay time (in ms) for the *j*-th stimulus displayed to the *i*-th participant, and the other terms can be interpreted as previously described. We fit the above model to the same three response variables (effective, genuine, and pleasant) using the same two-stage estimation procedure [29]. We used a cubic smoothing spline for the timing asymmetry effect function, and the three covariates were modeled as previously described, i.e., using a cubic smoothing spline for age and drinking, and a nominal smoothing spline for gender.

## Results

### Spatial properties

The AIC and BIC values for the fit models are given in Table 2. Note that both the AIC and BIC select Model 1 (from Table 1) as the optimal model for each of the three response variables. This implies that the perception of a smile involves a three-way interaction between the mouth angle, the smile extent, and the amount of dental show displayed in the expression. To quantify the model fit, we calculated the model coefficient of multiple determination (i.e., R-squared) without (*R*^2^) and with ($R_{*}^{2}$) the estimated random effect ${\hat{b}}_{i}$ in the prediction. We define *R*^2^ (or $R_{*}^{2}$) as the squared correlation between the response variable and the fitted values without (or with) the random effects included. In the leftmost columns of Table 3, we display the R-squared values from the optimal model, along with the estimated variance components. Table 3 reveals that the fixed-effects terms in the model explain about 10% of the variation in the response variables (i.e., $\text{cor}{\left(y_{ij},\hat{\eta }\left(x_{ij}\right)\right)}^{2}\approx 0.1$), whereas the model explains about 40% of the variation in the response variables with the random effects included (i.e., $\text{cor}{\left(y_{ij},\hat{\eta }\left(x_{ij}\right)+{\hat{b}}_{i}\right)}^{2}\approx 0.4$).

**Table 2:** Information criteria for the fit models.

	Effective		Genuine		Pleasant	
	AIC	BIC	AIC	BIC	AIC	BIC
1.	66.46	187.82	565.80	681.27	15.81	138.40
2.	131.40	236.67	603.65	714.47	72.20	173.13
3.	147.61	240.53	632.02	721.55	83.48	173.18
4.	154.31	240.31	648.83	730.57	84.52	170.05
5.	275.07	360.14	634.23	718.81	190.41	268.78
6.	184.12	259.47	696.93	779.78	114.31	192.81
7.	306.35	378.94	679.18	763.13	209.51	278.62
8.	305.46	371.27	685.56	752.07	217.78	283.36
9.	264.43	318.44	724.89	804.83	217.55	277.12

*Note*: A constant (i.e., 900) was added to each score.

**Table 3:** Model fit information for symmetric and asymmetric smiles.

	Symmetric Smiles			Asymmetric Smiles		
	Effective	Genuine	Pleasant	Effective	Genuine	Pleasant
*R*^2^	0.0981	0.0718	0.1247	0.0399	0.0404	0.0660
$R_{*}^{2}$	0.4664	0.3911	0.3902	0.6346	0.6177	0.6914
*σ*^2^	0.0413	0.0507	0.0405	0.0374	0.0456	0.0349
*θ*^2^	0.0124	0.0098	0.0076	0.0094	0.0102	0.0130
*ρ*	0.2314	0.1626	0.1589	0.2006	0.1830	0.2717

*Note 1*: Symmetric Smiles results are those for Model 1.

*Note 2*: *ρ* = intra-class correlation coefficient: *θ*^2^/(*θ*^2^ + *σ*^2^).

**Fig 6:**
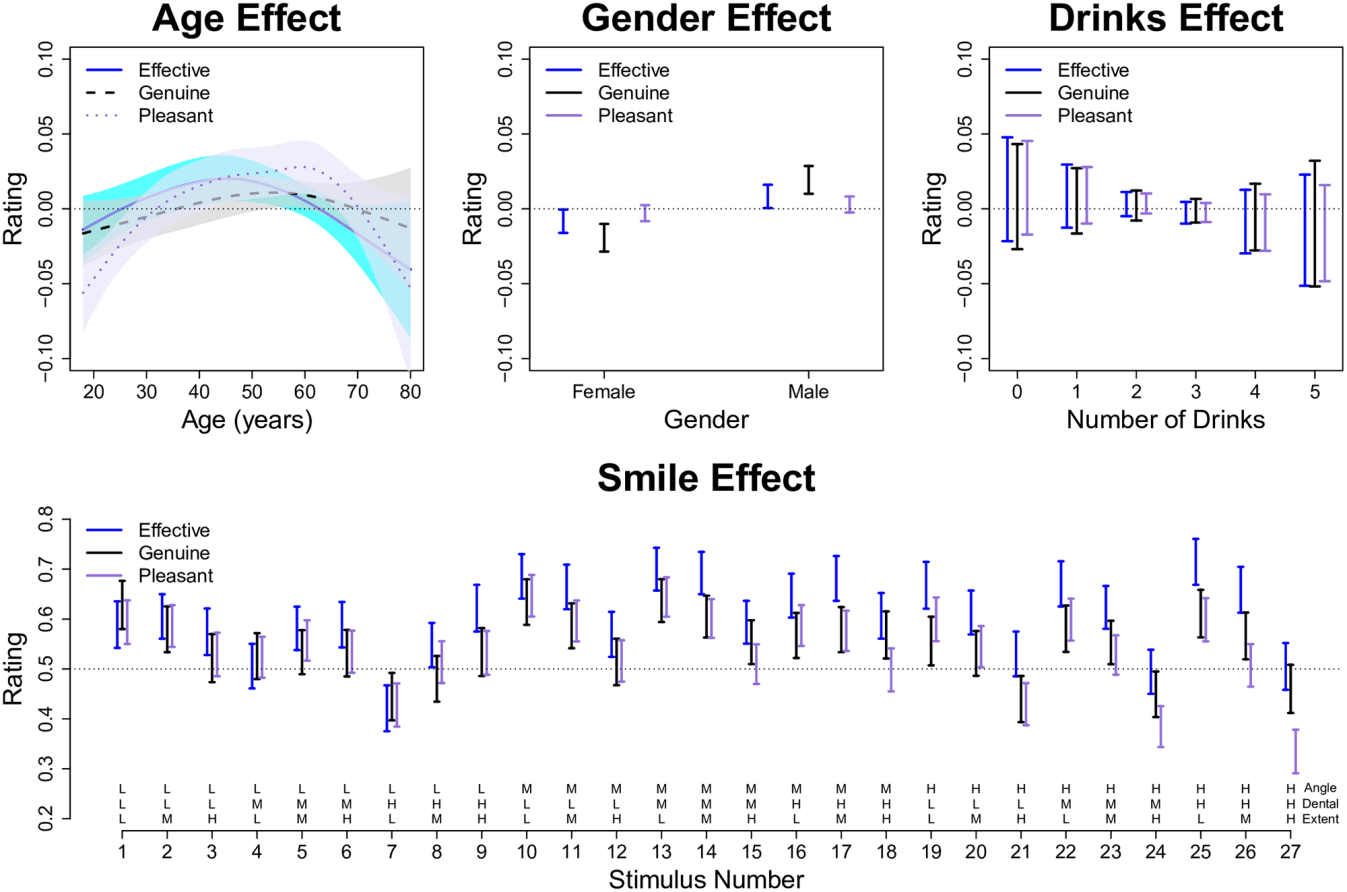
Predictions for SSANOVA model of symmetric smiles. The top row plots the estimated main effect functions for the three covariates: age, gender, and drinking. The bottom row plots the estimated smile effect function predictions for each of the 27 smile animations depicted in Fig 1. Within each subplot, the shaded regions or bars denote 90% Bayesian confidence intervals.

We plot the SSANOVA model predictions (i.e., estimated effect functions) for the optimal model in Fig 6. The main effect functions for age (Fig 6, top left) reveal that there is quadratic trend such that younger and older participants give slightly lower ratings; however, the confidence intervals on the age effect functions are rather wide and the trend is not significantly different from zero for two of the three response variables. Similarly, we see little to no effect of gender (Fig 6, top middle) or the number of alcoholic drinks (Fig 6, top right). In contrast, the smile effect function (Fig 6, bottom) reveals that (i) aside from the extreme smiles with high angle and high extent, the ratings of the three response variables are rather similar within each smile, and (ii) there are significant differences between the ratings across the 27 smiles.

**Fig 7:**
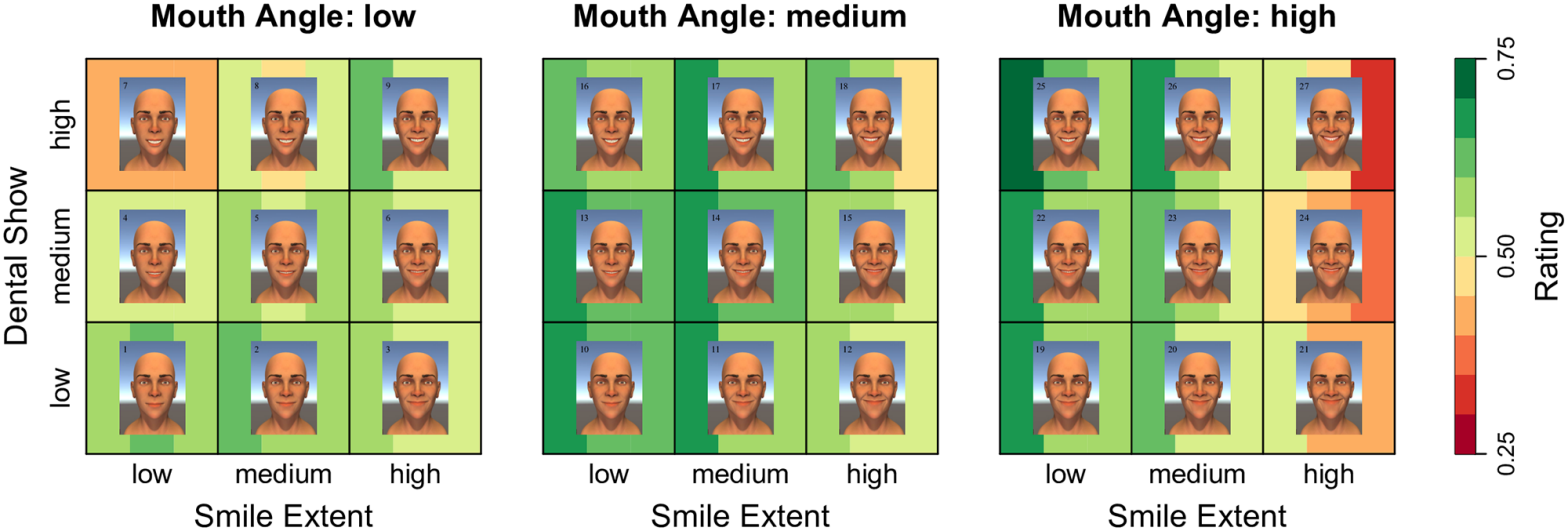
Visualization of the smile effect. A heat-map plotting the three-way interaction between the smile parameters. The three vertical bars behind each face denote the predicted score for the three response variables: effective, genuine, and pleasant (respectively). Greener colors correspond to better (i.e., higher rated) smiles, and redder colors correspond to worse (i.e., lower rated) smiles.

To obtain a better understanding of the three-way interaction effect, Fig 7 plots the smile effect as a function of three factors (angle, extent, dental show). This plot reveals that there is a sweet spot of parameters (particularly mouth angle and smile extent) that results in the most successful smiles. The highest rated smiles were those with low to medium extents in combination with medium to high angles. Using the parameter definitions in Fig 2, successful smiles have mouth angles of about 13–17° and smile extents of about 55–62% the interpupillary distance (IPD). However, as is evident from Figs 6 and 7, the best smiles represent a diverse collection of different combinations of facial parameters. This reveals that, although there is an optimal window of parameters, there is not a single path to a successful smile.

**Fig 8:**
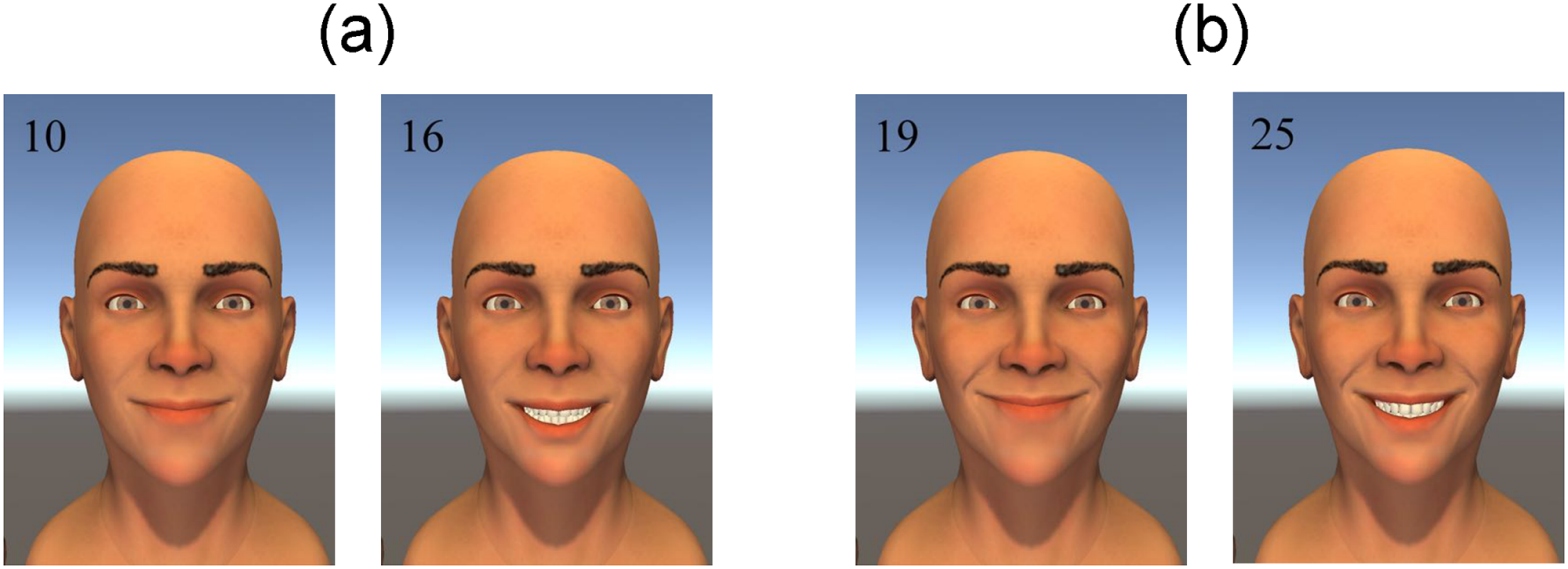
Dental show effect at different levels of angle-extent. (a) Two smiles with smaller angle-extent combinations. (b) Two smiles with larger angle-extent combinations. Increasing dental show makes the smile worse (i.e., less successful) for (a) and better (i.e., more successful) for (b).

Fig 7 also reveals that there are particular combinations of the smile parameters that result in unsuccessful smiles. One interesting finding was how low the ratings were for smiles with extreme angles. Another interesting finding is that the effect of dental show on the smile ratings differs depending on the angle-extent combination of the smile. For smiles that have smaller angle-extent values, displaying low or medium dental show is better than displaying high dental show. In contrast, for smiles with medium to large angle-extent combinations, displaying high dental show is better. This point is illustrated in Fig 8. However, for smiles with angle-extent combinations that are too large (i.e., smiles 21, 24, 27), increasing dental show decreases smile quality.

**Fig 9:**
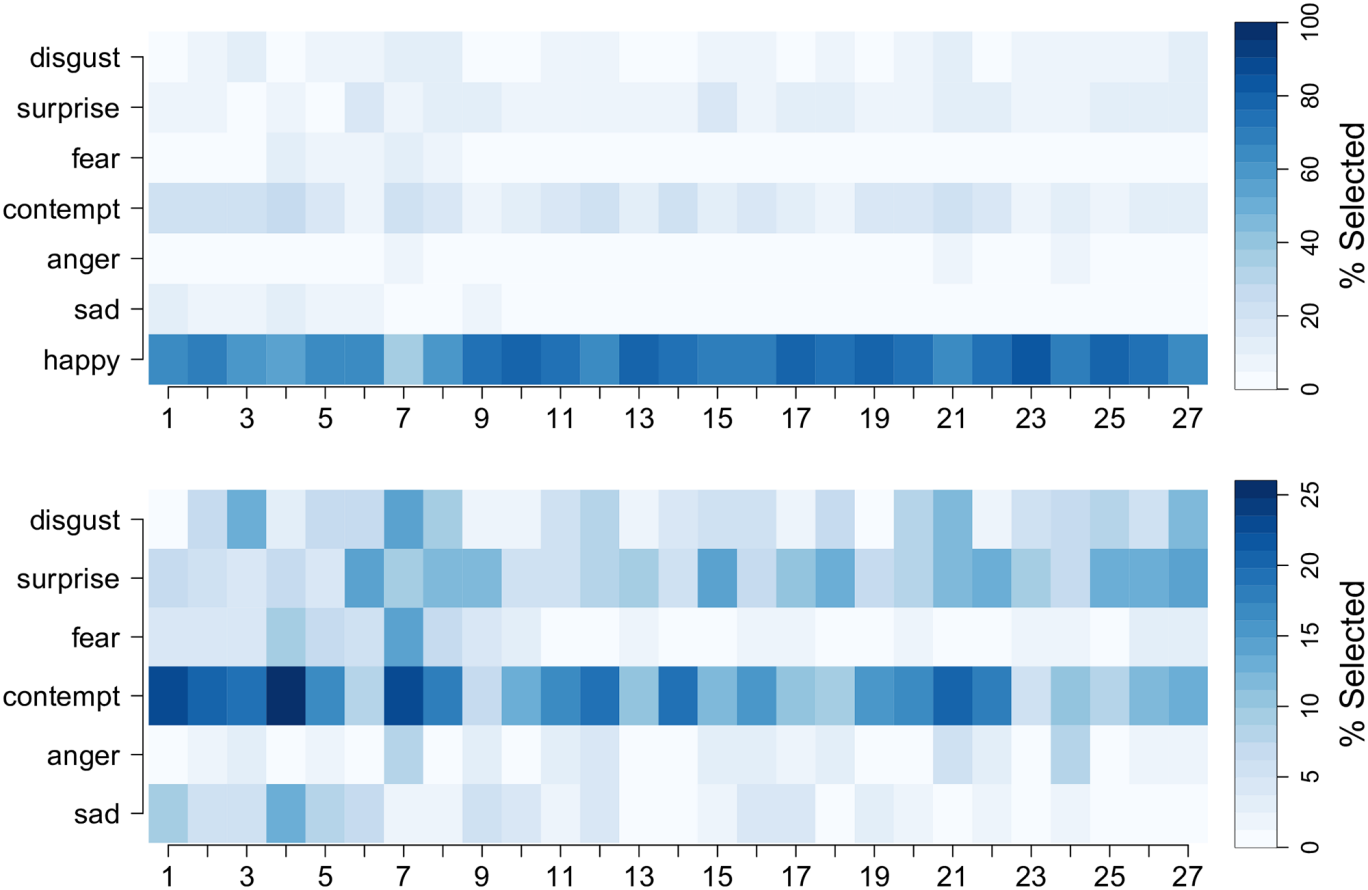
Perceived emotions for the 27 smiling faces. The percentage of subjects who selected the given emotion (rows) for each smile (columns). The top subplot depicts the results for all seven emotions, whereas the bottom subplot provides a more detailed look at the non-happy emotions that were perceived from each expression.

To understand which emotions were perceived from each smile, Fig 9 plots the percentage of participants who selected each of the seven emotions for each of the 27 smiles. From the top subplot of Fig 9, it is evident that the emotion “Happy” was selected most often—which was expected. To better visualize which non-happy emotions were perceived from the smiles, the bottom subplot of Fig 9 shows the percentage of participants who selected the six non-happy emotions. This plot reveals that (i) “Contempt” is the most frequently perceived non-happy emotion, (ii) participants tended to perceive “Contempt” from a variety of different types of smiles, and (iii) smiles with a combination of low angle and low extent showed the largest percentages of “Contempt” ratings.

### Temporal properties

**Fig 10:**
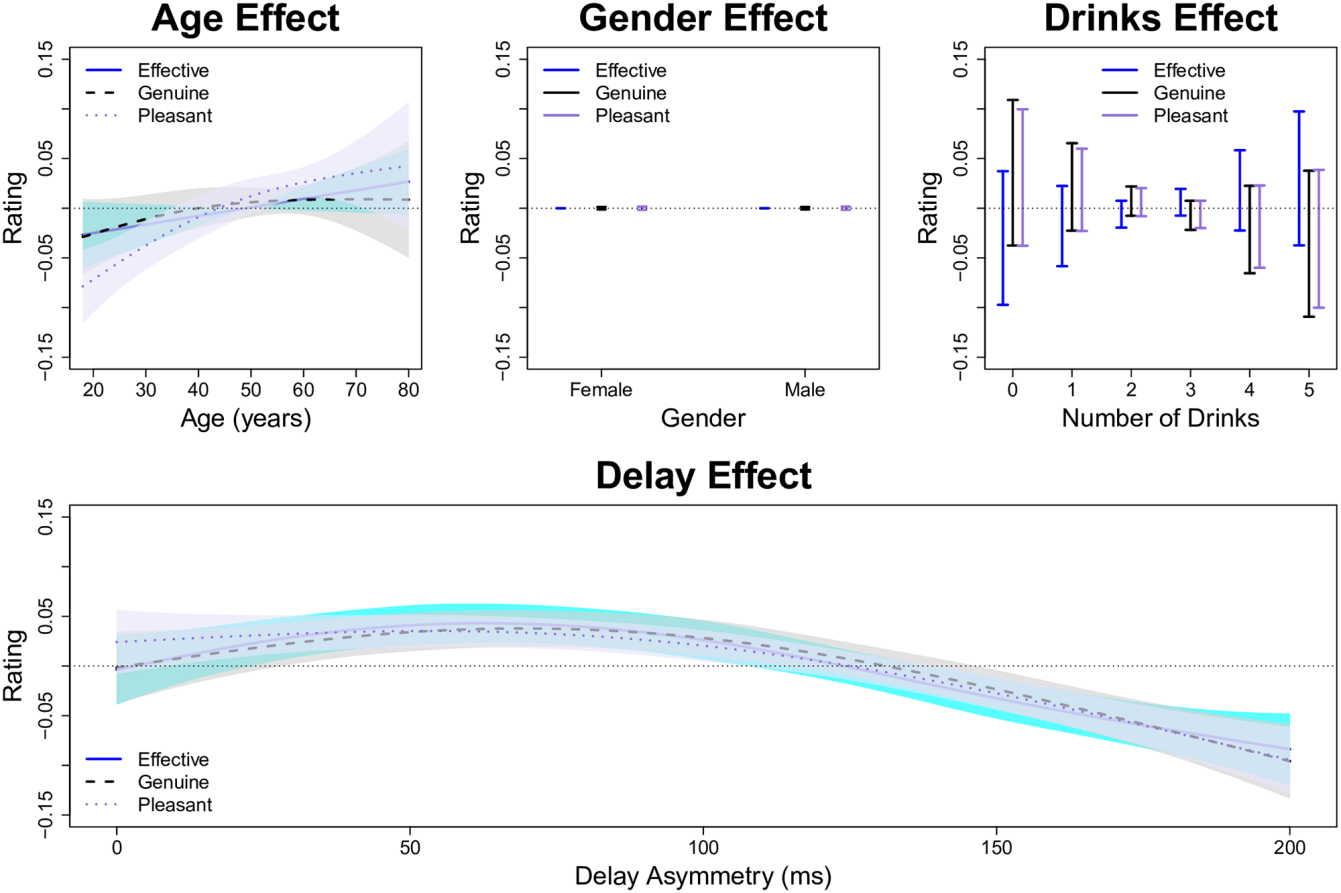
Predictions for SSANOVA model of asymmetric smiles. The top row plots the estimated main effect functions for the three covariates: age, gender, and drinking. The bottom row plots the estimated timing asymmetry effect function. Within each subplot, the shaded regions or bars denote 90% Bayesian confidence intervals.

The fit statistics for the SSANOVA models fit to the asymmetric smiles are given in the rightmost columns of Table 3. Note that the models explained about 4% of the data variation at the aggregate level and about 60% of the variation at the individual level. The SSANOVA model predictions (i.e., effect functions) are plotted in Fig 10. In this case, we see that there is a trend such that older participants provide higher ratings (Fig 10, top left); however, the confidence intervals are wide, and the trend is insignificant for two of the three predictors. Similar to the previous model, there is no significant gender effect (Fig 10, top middle) or drinking effect (Fig 10, top right). The interesting result from this model is plotted in the bottom portion of Fig 10. We find that having a slight asymmetry (25–100 ms) increases the smile ratings by a significant amount compared to having a perfectly symmetric smile. However, a delay asymmetry of 125 ms or more resulted in reduced smile ratings, which decreased almost linearly with the delay asymmetry in the range of 100–200 ms. At the largest delay asymmetry (200 ms), the expected smile ratings were about 0.09 units below the symmetric smile ratings.

## Discussion

Our results shed new light on how people perceive dynamic smile expressions. Using an anatomically-realistic 3D facial tool, we determined which spatial (angle, extent, dental show) and temporal (delay asymmetry) smile parameters were judged to be successful by a large sample of participants. Most importantly, our results allow us to both dispel and confirm commonly held beliefs in the surgical community, which are currently guiding medical practice. Our result regarding the optimal window (or sweet spot) of smile extent contradicts the principle that “more is always better” with respect to smile extent. Consequently, using absolute smile extent (or excursion) as a primary outcome measure—as is currently done in practice [14, 43]—is inappropriate. Instead, clinicians should use both mouth angle and smile extent as outcome measures because an effective smile requires a balance of both.

Among medical professionals, there is a debate about the importance of showing teeth during smiling, with some believing it to be of paramount importance while others trivialize its role. Our finding that dental show significantly influences the perception of a smile clarifies this debate. Specifically, the degree of dental show can have negative or positive effects: increasing dental show can decrease smile quality (for low angle-extent smiles), increase smile quality (for high angle-extent smiles), or have little influence on smile quality (for medium angle-extent smiles). Thus, the interaction between dental show and the angle-extent parameters confirms the idea that individuals with limited facial movement should be encouraged to form closed-mouth smiles (see Fig 8). Our results reveal that forming open-mouth smiles with small angles/extents can produce unintended perceptions of the expression, e.g., contempt or fear instead of happiness.

Our finding that small timing asymmetries can increase smile quality may seem counter-intuitive, in light of past research revealing that people tend to prefer symmetric faces [44, 45]. However, this result is consistent with principles of smile design in which dynamic symmetry (i.e., being very similar but not identical) “allows for a more vital, dynamic, unique and natural smile” compared to static symmetry (i.e., mirror image), see [46, pg. 230]. Furthermore, this finding is consistent with some research which has found that slightly asymmetric faces are preferred over perfectly symmetric faces [47–49]. Our results suggest that this preference relates to the perception of the genuineness and pleasantness of the smile expression, such that slight timing asymmetries are viewed as more genuine/pleasant (see Fig 10).

Our discovery of the threshold at which delay asymmetries become detrimental to smile quality (i.e., 125 ms) provides a helpful benchmark for clinicians and therapists. This finding compliments past research which has found that emotional cues can be perceived within 100–200 ms of encountering an image of a face [4, 5]. The smile is successful long as the left-right smile onset symmetry remains within 125 ms. Beyond 125 ms, delay asymmetries have a noteworthy negative effect such that a 200 ms delay asymmetry results in an expected 0.09 unit decrease in smile ratings. Note that this decrease is a medium effect size with respect to psychological standards [50]: defining $\hat{d}={\hat{\eta }}_{T}\left(200\right)/\hat{\sigma }$, we have that $\hat{d}=-0.43$ for effectiveness, $\hat{d}=-0.45$ for genuine, and $\hat{d}=-0.51$ for pleasant. Furthermore, the difference between the ratings with a 75 ms versus a 200 ms delay is a medium-large effect size by typical psychological standards: defining $\hat{d^{*}}=\left[{\hat{\eta }}_{T}\left(75\right)-{\hat{\eta }}_{T}\left(200\right)\right]/\hat{\sigma }$, we have that $\hat{d^{*}}=0.65$ for effectiveness, $\hat{d^{*}}=0.62$ for genuine, and $\hat{d^{*}}=0.68$ for pleasant. It is interesting to note that modifying only the dynamic symmetry can have such a noticeable effect on how the smile is perceived.

In summary, our findings complement the literature on the dynamics of facial expressions of emotion [15, 25]. Similar to past studies [16–18], we have found that computer generated models of facial expressions can be a useful tool for systematically studying how people perceive facial expressions of emotion. Our results agree with past literature that has found dynamic (spatiotemporal) aspects of facial expressions to be important to their perception [19–24]. In particular, we found that a successful smile consists of (i) an optimal window of mouth angle and smile extent, (ii) the correct amount of dental show for the given angle-extent combination, and (iii) dynamic symmetry such that the left and right sides of the mouth are temporally synced within 125 ms. Consequently, our results extend the literature by providing spatiotemporal benchmarks of a successful smile with respect to clinically meaningful parameters.

## Conclusion

Our study looked at how dynamic (spatiotemporal) properties of mouth movement relate to perceptions of facial expressions generated by a 3D computer model. We found that a successful smile involves an intricate balance of mouth angle, smile extent, and dental show in combination with dynamic spatiotemporal timing. Future research should encompass more combinations of angle, extent, dental show, and timing parameters, in order to develop a more complete spatiotemporal understanding of how the interplay between these elements affects individuals’ perceptions of the smile trajectory. Also, future studies could consider manipulating additional facial features (e.g., orbicularis oculi contraction) to create a more diverse set of facial expressions. Additionally, 3D cameras could be used to create scans of people smiling to enable the data-driven generation of emotional expressions, replacing the artist-created blend shapes approach used in our study [26, 51, 52]. Such an approach could be useful for fine-tuning the smile stimuli used in this study, which have the limitation of being artist-generated. Furthermore, 3D cameras could be used to study timing asymmetries in a more diverse sample of facial expressions, which would be useful for examining the robustness of our timing asymmetry effect. Note that our results regarding timing asymmetries have the limitation of coming from a single smile expression (i.e., smile 22). Another useful extension of our study would be to examine how a large sample of participants perceive a variety of other facial expressions of emotion, e.g., surprise, anger, fear, or sadness. Finally, the integration of biologic measurements (e.g., eye-tracking or electroencephalography) could provide useful data about the perception of dynamic facial expressions.

## Supporting information

S1 Dataset Smile ratings. Data and R code to reproduce results.
(ZIP)

S1 Video Smile videos. Smile animations (stimuli) used in paper.
(ZIP)
